# The *Plasmodium vivax* in China: decreased in local cases but increased imported cases from Southeast Asia and Africa

**DOI:** 10.1038/srep08847

**Published:** 2015-03-05

**Authors:** Jun Feng, Huihui Xiao, Li Zhang, He Yan, Xinyu Feng, Wen Fang, Zhigui Xia

**Affiliations:** 1 National Institute of Parasitic Diseases, Chinese Center for Disease Control and Prevention; Key Laboratory of Parasite and Vector Biology, MOH; WHO Collaborating Centre for Malaria, Schistosomiasis and Filariasis; Shanghai, People's Republic of China

## Abstract

Currently the local *P. vivax* was sharply decreased while the imported vivax malaria increased in China. Despite Southeast Asia was still the main import source of vivax malaria, the trend of Africa become serious, especially for west and central Africa. Herein we have clarified the trend of *P. vivax* in China from 2004–2012, and made some analysis for the differences of imported vivax back from different regions. There are significantly different of *P. vivax* between Southeast Asia and Africa, also the difference was observed for different regions in Africa. Additionally, we have explored the possibility for the difference of the *P. vivax* between migrant workers back from west and central Africa and the prevalence of local population. This reminds us that surveillance and training should be strengthened by medical staffs on the imported *P. vivax* cases reported especially from west and central Africa, in order to reduce the risk of malaria reintroduction and, specific tools should be developed, as well as the epidemiological study to avoid the misdiagnosis such as *P. ovale* and *P. vivax*.

P*lasmodium vivax* is the most common and has the widest geographic range of the four human malaria species. It was mostly found outside of Africa and especially prevalent in Southeast Asia and America^1^. *P. vivax* caused considerable morbidity despite less mortality than *P. falciparum*. These two species coexist in many parts of tropical regions, except west and central Africa where *P. vivax* was almost absent^2^. This is mainly due to the high prevalence of the Duffy negative phenotype, caused by a single nucleotide polymorphism in the erythroid-specific promoter region of the DARC (Duffy Antigen Receptor for Chemokines) gene, occurs in over 95% of the population of west and central Africa and confers complete protection against *P. vivax* malaria^3^.

In China, *P. vivax* was the major species for relatively long time. During 2004 to 2012, it account for 76.9% of all reported malaria cases and reach the peak in 2006^4^,^5^,^6^,^7^,^8^,^9^,^10^, particularly in central China such as Anhui and Henan provinces where *Anopheles sinensis* mosquitoes was prevalent^11^,^12^,^13^.

Since 2010, China has initiated National Malaria Elimination Action Plan (NMEAP)^14^, the local *P. vivax* was decreasing continuously, but due to the increasing investment and numbers of Chinese laborers working abroad, the proportion of imported *P. vivax* was up for recent years. For example, 1,183 imported *P. vivax* cases were reported in 2011, which accounts for 57.0% of the total *P. vivax* cases. The imported *P. vivax* malaria may bring out the high risk to the malaria-free localities in which *Anopheles sinensis* mosquitoes are prevalent during the transmission season.

Currently, the trend of *P. vivax* cases imported from Southeast Asia and Africa was increased, especially for west and central Africa. Despite its rare reported according to the WHO report^15^, the *P. vivax* malaria was frequent identified among the migrant workers back from these areas in China. Herein we have clarified the trend of *P. vivax* in China from 2004–2012, and made some analysis for the differences of imported vivax back from different regions.

## Results

### Prevalence of *P. vivax* in China, 2004–2012

**Figure 1:**
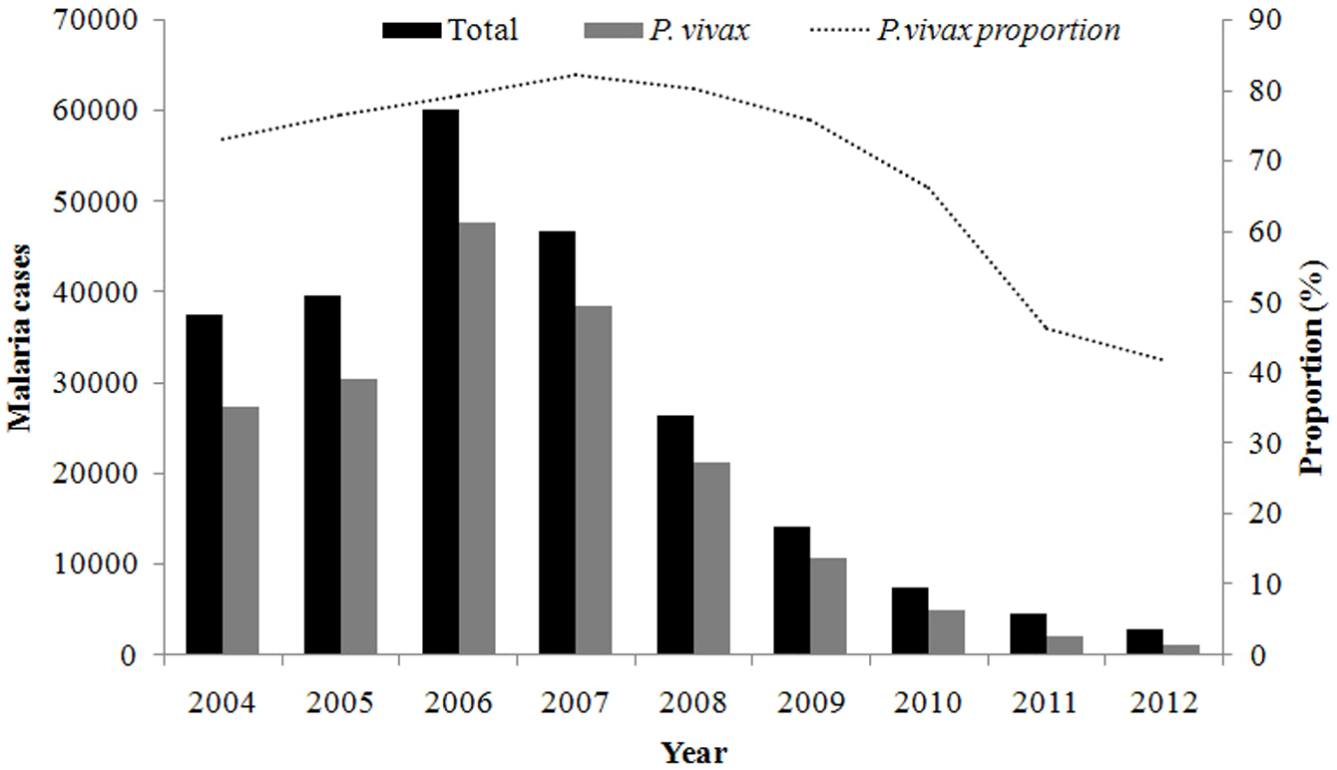
The prevalence of *P. vivax* malaria in China, 2004–2012. The lines and columns of different colors represent the change trend of total malaria cases (black), *P. vivax* cases (grey) and *P. vivax* proportion in the all reported malaria cases (dash line), respectively.

Totally 239,111 malaria cases were reported during 2004 to 2012 and *Plasmodium vivax* (n = 183,902) accounting for 76.9% of all reported cases in China. During this period, the majority of *P. vivax* malaria was local cases, increased to peak in 2006, particularly in central China such as Anhui and Henan provinces where *Anopheles sinensis* mosquitoes was prevalent (Figure 1). Since 2007, vivax malaria declined and the proportion of *P. falciparum* increased significantly.

After NMEAP launched in 2010, each malaria cases was then required to be investigated and identified as indigenous or imported, so since then every *P. vivax* case could be distinguished^16^. Though a 57.7% decrease in total *P. vivax* cases from 2010 (n = 4,901) to 2011 (n = 2,075), the proportion of imported *P. vivax* was has increased from 31.5% to 57.0%.

**Figure 2:**
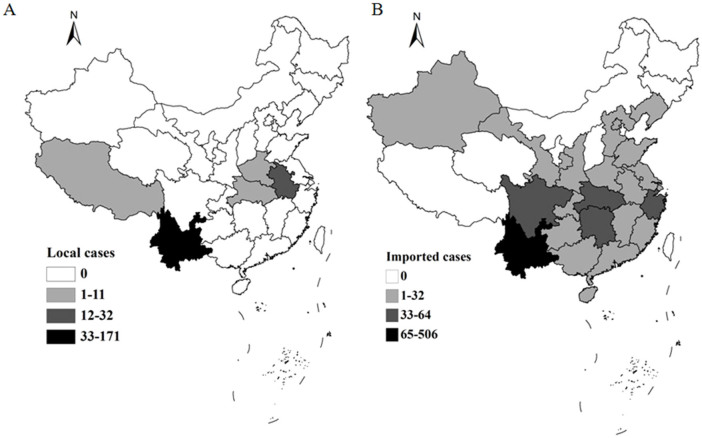
Distribution of local and imported *P. vivax* in China, 2012. A) Local *P. vivax* cases; B) Imported *P. vivax* cases. The map was created using ArcGIS 10.1.

**Figure 3:**
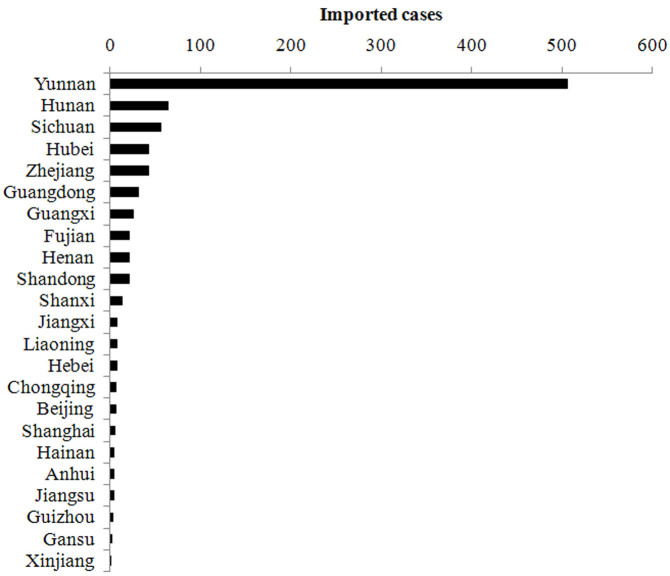
Provincial distribution of imported *P. vivax* in China, 2012.

In 2012, a total of 1,143 *P. vivax* malaria cases were reported, responsible for 41.9% of the total malaria cases. The imported and local *P. vivax* was 909 and 234, taking up 79.5% and 20.5% of total *P. vivax* malaria cases, respectively. The local *P. vivax* was mainly occurred in Yunnan province (n = 171 [73.1%]), particularly in the counties around the China-Myanmar border (Figure 2A)^17^. The imported *P. vivax* malaria was spread to most regions of China (23 provinces), among them, Yunnan (n = 506 [55.7%]), Hunan (n = 64 [7.0%]) and Sichuan (n = 56 [6.2%]) had the largest number of imported vivax malaria cases (Figure 2B). The common characterize of these provinces was imported vivax back from Southeast Asia, account for the most source of total *P. vivax*. For example, vivax cases from Southeast Asia into top five provinces including Yunnan, Hunan, Sichuan, Hubei and Zhejiang responsible for 96.0% (486/506), 76.6% (49/64), 58.9% (33/56), 55.8% (24/43) and 66.7% (28/42) of total imported vivax cases, respectively (Figure 3).

### Difference between *P. vivax* from Africa and Southeast Asia

Migrant workers coming back from Southeast Asia were the most imported source of *P. vivax* in China, and this was significantly different from those coming back from Africa (χ^2^ = 869.2063, p < 0.0001) in 2012 (Table 1). Totally 697 vivax cases were reported from Southeast Asia and the cases were mainly imported from Myanmar (n = 557 [59.3%]), Cambodia (n = 46 [4.9%]) and Laos (n = 32 [3.4%]). However, the *P. vivax* cases only represent 13.2% (n = 192) of all cases returning from Africa, and Equatorial Guinea (n = 25 [1.7%]), Ethiopia (n = 21 [1.4%]), and Ghana (n = 21 [1.4%]) were the main imported source.

### Difference between *P. vivax* from different regions of Africa

The *P. vivax* imported from different regions of Africa also varied. Regarding to west Africa, it was differing from other regions of Africa (Table 1). In 2012, *P. vivax* account for 9.5% (n = 61) by west Africa, while this figure was 16.2% (n = 131, p = 0.0007) in other regions of Africa. The difference was also observed among the migrant workers from west Africa and central Africa (χ^2^ = 29.7962, p = 0.0063), east Africa (χ^2^ = 33.9816, p = 0.0002) and north Africa (χ^2^ = 64.2363, p = 0.0002) (Table 1). Surprisingly, there is little difference between the migrant workers from west Africa and south Africa (p = 0.2043), where the *P. vivax* constituent ratio in south Africa was 9.2% (n = 17).

### Difference between *P. vivax* in migrant workers and local population in west and central Africa

Surveillance of malaria cases imported into China in 2012, reveals that 122 cases of *P. vivax* originated from 21 west and central African countries. In the same time period, there were 988 cases of imported *P. falciparum* from the same countries. This gives a surprisingly high ratio of 100:12 *P. falciparum* to *P. vivax* infections coming back from these areas (Table 2). In addition, low *P. vivax* prevalence was observed in west and central Africa because of the high prevalence of Duffy negative phenotype in the local populations, so comparing with this, the imported *P. vivax* into China from these regions was extremely high, which has aroused our interesting of the difference.

## Discussion

*P. vivax* was the major species in China for a long time and was responsible for more than 90% of the local malaria^18^. Vector control and drug treatment of febrile individuals had been highly successful in the endemic areas in China^19^. However, *P. vivax* had outbreaks frequently in many counties of central provinces and serve as the predominant parasite species around 2006^12^. To solve this, the government has launched the National Malaria Control Program (2006–2015) and National Malaria Elimination Action Plan (2010–2020) to strengthen malaria prevention and control at all levels, therefore the epidemics have well controlled^14^,^20^.

However, due to the increasing Chinese investment and numbers of laborers working abroad, it may bring out high risks from malaria-endemic areas such as Africa and Southeast Asia on malaria morbidity in non-endemic regions of China^21^. For example, it was estimated to be 40.8 billion investment and 22,717 million laborers in Africa in 2012, respectively; and these were increased by 13.0% and 38.3%, respectively, from numbers in 2011. Though Southeast Asia, particular Myanmar was still the main imported source of *P. vivax* malaria, patients from Africa especially west and central Africa was increased significantly. For instance, the proportion of vivax malaria from west and central Africa was only account for 2.1% (n = 32) of imported *P. vivax* cases in 2010, but in 2012, this increased to 13.4% (n = 122) (Table 3). This may bring out the retransmission risk to the endemic areas where the suitable *Anopheles sinensis* are prevalent. Since little was known about the difference of the imported *P. vivax* by acquisition, here we characterize the *P. vivax* trend in China from 2004 to 2012, and made some analysis for the differences of imported vivax back from Southeast Asia and Africa in 2012. In this text, not every case could be distinguished as being indigenous or imported before 2010, because the data were not available. Only since the NMCAP launched in 2010, each malaria cases was then required to be investigated and identified as indigenous or imported.

Data showed Southeast Asia was still the major imported source of *P. vivax*, while migrant workers coming back from Africa were increased recently. The geographical distribution of *P. vivax* within Africa is patchy, with sporadic areas of transmission scattered throughout the continent, particularly extremely rare in west and central Africa, with the exception of the horn of Africa and other areas such as island of Sao Tome, which is known to harbour all four human malaria parasites^22^. Though *P. vivax* malaria was rare reported in west and central Africa, migrant workers consistently return to China with vivax malaria was detected after visiting these regions, this was more obviously since 2010. We have wondered why the significant difference occurred between the imported *P. vivax* data and the low prevalence local population.

Rare accounts detailing *P. vivax* infections in non-African travellers returning from these areas^23^. For example, imported malaria surveys from the USA showed the data of 2004 revealing that 65% of *P. vivax* imported into the USA from Africa in that year (n = 67) originated in countries in west and central regions^24^. Furthermore, 49 *P. vivax* cases were detected from the travelers coming back from Nigeria, Cameroon, Liberia, Republic of Congo/Democratic Republic of Congo and Ghana during 2006 to 2011^25^,^26^,^27^,^28^,^29^,^30^.

It was found that the difference mainly associated with human populations in which the Duffy negative phenotype is present at a lower frequency than elsewhere^23^. Migrant workers or non-African expatriates may preferentially visit areas of west and central Africa where there is a relatively high frequency of Duffy positive individuals in the local population and where *P. vivax* is more likely to be transmitted.

However, recent report has showed that *P. vivax* is endemic in west and central Africa for it has been identified in both chimpanzees and gorillas from this region through mitochondrial genome sequencing^31^,^32^,^33^,^34^. This genetically based evidence may explain at least in part, for the presence of *P. vivax* among the largely Duffy negative human populations of west and central Africa. Another report indicated that *P. vivax* transmission can be expected in populations with high levels of RBC Duffy negativity and in which malaria transmission intensities are sufficiently high, as is the case in many areas of west and central Africa^35^. The capacity of the environments in these regions to transmit malaria is so high that even a very small proportion of the human population of the Duffy positive phenotype could sustain the presence of *P. vivax*^36^.

Another factor that may contribute to this difference is the higher transmissibility of *P. vivax* under adverse conditions^37^. Features of the transmission biology of *P. vivax* give this species greater resilience than the less robust *P. falciparum* in the face of conditions adverse to the transmission of the parasites. This may lead to the consequence that there should be higher proportion of *P. vivax* in the vector mosquitoes than there is in the corresponding human population. Then the travellers, a probe of the infection rates in the local mosquitoes, can be expected to have higher proportions of *P. vivax* than are found in the endemic human populations. Similarity, recent finding of Ryan indicated the presence of *P. vivax* in 0.65% of mosquitoes from an area of west Kenya in the local population^38^.

The misuse of prophylactic anti-malaria drugs among travellers may also contribute to this phenomenon. According to the antimalarial drug policy of China, chloroquine was recommended antimalarial chemoprophylaxis used in the *P. vivax* endemic area, and piperaquine phosphate was recommended antimalarial chemoprophylaxis used in the mixed *P. falciparum* and *P. vivax* endemic area. However, the Chinese travelers often take artemisinin combined therapies (ACTs) to treat with malaria in Africa, and even many travelers are not taking the drugs during the radical treatment, therefore it will not protect against relapses after cessation of drug use. This is also true of *P. ovale*, which is also capable of producing hypnozoites, and may explain the higher rate of this parasite in returning travellers compared to the local populations (Table 2).

It is also probable that a small proportion of imported cases may be *P. ovale* infections rather than *P. vivax*, as is often difficult to distinguish the two species by microscopy^39^. Despite 117 *P. vivax* cases (95.9%) were laboratory-confirmed including microscopy, PCR and Rapid Diagnostic Tests (RDTs), the cases detected by microscopy took up a large proportion. Though these cases were all re-confirmed using PCR by reference labs in the provincial CDC, it should continued to develop more accurate and specific skills detecting these two species, currently some accurate molecular detection systems were developed for imported *P. ovale* with genetic variations *P. ovale wallikeri* and *P. ovale curtisi*^40^.

In a word, the present study indicates that frequent identification of *P. vivax* in travelers coming back from west and central Africa in China, despite the prevalence of this parasite in local populations is relatively low. The imported *P. vivax* could be introduced into malaria-free localities during the transmission season, especially when a large number of cases are clustered in areas where *Anopheles sinensis* are prevalent. Though there are no secondary transmission of imported *P. vivax* in China, surveillance systems should also be strengthened to ensure timely recognition and prompt response for effective management. More attention should be paid by Chinese medical staffs on the imported *P. vivax* cases reported especially from west and central Africa. First, they should raise awareness that *P. vivax* could be imported from these regions. Second, training should also be strengthened and more accurate, specific tools should be developed, as well as the epidemiological study to avoid the misdiagnosis between *P. ovale* and *P. vivax*. Only the epidemiology and molecular study have proved the *P. vivax* was imported from west and central Africa, and this may indicated that DARC is only a relative resistance factor and *P. vivax* must be circulating at low concentrations in west and central Africa.

## Methods

### Study Design

Studied population was all patients with parasitological diagnosed for vivax malaria between 2004 and 2012 in China. For the analysis of *P. vivax* malaria trend, data from 2004 to 2012 were collected, reviewed and analyzed. For the analysis of the discrepancy of *P. vivax* distribution by acquisition, the individual *P. vivax* case from the web-based reporting system in 2012 was carefully characterized. These cases were clinical diagnosed or laboratory-confirmed *via* microscopy, PCR and RDTs. In this analysis, an imported case of malaria was defined as case of malaria acquired in a known malarious area outside China. In China, the following criteria for imported malaria must be simultaneously met: 1) the patient was given a diagnosis of malaria; 2) the patient had a travel history to malaria-endemic areas outside China during malaria transmission season; and 3) the onset time for malaria for the person was <1 month after returning to China during the local transmission season. This definition of malaria was based on the latent period for all *Plasmodium* species reported in China. The map of local and imported *P. vivax* malaria distribution in China was created by ArcGIS 10.1 (Environmental Systems Research Institute, Inc.).

### Data collection

All individual cases from the web-based reporting system (China Information System for Diseases Control and Prevention V2.0) were carefully reviewed and analysed. In China, malaria is a statutory notifiable infectious disease, and cases are identified according to the unified diagnostic criteria issued by the Chinese Ministry of Health, which includes the definitions of clinically diagnosed and laboratory-confirmed cases. Clinically diagnosed cases were defined as a patient with malaria-like symptoms, having lived in or recently travelled to areas with known malaria endemic areas. Laboratory confirmed cases were defined as clinically diagnosed cases with microscopy, rapid diagnostic tests (RDTs), or polymerase chain reaction test. Health workers in both the public and private medical sectors were required to report malaria cases. The data was selected by dates of onset, reporting area and final review. This collecting type could cover all the *P. vivax* cases reporting during January 1^st^ to December 31^st^ of 2012. For the local and imported data, we collected it through annual malaria statistics reporting system.

### Statistical analysis

A descriptive analysis was processed using Microsoft Excel and SAS software (SAS INSTITUTE INC., Version 9.2). The chi-square test (χ^2^) or Fisher's exact test was used to investigate whether the constitute ratio of *P. vivax* have the statistically significant difference between two groups (Africa and Southeast Asia, and different regions of Africa). The p values was calculated, in order to reduce the probability of false positive errors and considered statistically significant when <0.01.

## Author Contributions

J.F. conceived the study, and drafted the manuscript. H.H.X., W.F. undertook statistical analysis. L.Z., H.Y. and X.Y.F. analysed the data and gave some good suggestions on the manuscript. Z.G.X. initiated the study and made major contributions to drafting the manuscript. All authors contributed to the writing of the manuscript and approved the submitted version of the manuscript.

## Figures and Tables

**Table 1:** Parasite species imported into China from other regions in 2012

Regions	Total	*P. vivax*	*P. falciparum*	*P. malariae*	*P. ovale*	Mix	Unclassified
West Africa	641	61	554	6	10	5	5
Central Africa	537	61	434	8	25	6	3
South Africa	35	4	29	1	1	0	0
North Africa	70	19	51	0	0	0	0
East Africa	97	31	64	0	2	0	0
Southeast Asia	940	697	228	3	2	9	1

**Table 2:** Parasite species prevalence of traveler's malaria imported into China from west and central Africa compared to that of the local populations

	Total cases	
Species	Imported to China 1	Local populations
*P. vivax*	122	NA 2
*P. falciparum*	988	Almost
*P. malariae*	14	NA 2
*P. ovale*	35	NA 2

^1^Data were from the current survey.

^2^NA represent that no countries were found the prevalence of *P. vivax*.

**Table 3:** Migrant workers of imported *P. vivax* malaria back to China from west and central Africa, 2010–2012

Year	Total imported *P. vivax*	Africa	West Africa	Central Africa
2010	1542	64	19	13
2011	1183	138	52	45
2012	909	192	61	61
